# Editorial: Bioluminescent indicators and sensors for biomedicine and environmental analysis

**DOI:** 10.3389/fbioe.2022.1057607

**Published:** 2022-11-17

**Authors:** Vadim R. Viviani

**Affiliations:** Department Physics, Chemistry and Mathematics, Center for Sustainable Sciences and Technologies (CCTS), Federal University of São Carlos, Sorocaba, Brazil

**Keywords:** bioluminescence, biosensors, bioimaging, reporter genes, smartphone CCD imaging, near-infrared bioluminescence

At times in which the biosphere and humanity are undergoing major changes, bioanalytical and bioindication techniques to monitor health, the environment and emerging threats are on high demand. Bioluminescence, the emission of visible light by living organisms for communicative purposes, has been extensively used for bioanalytical purposes to assess biological and environmental integrity, alerting humanity about biological and environmental threats, and assessing industrial products microbiological quality. In this Research Topic, some of the latest advances about the improvement of bioluminescence systems and of associated detecting technologies for biomedical, environmental and safety bioanalysis purposes are reported.

• The increase of sensitivity of smartphone cameras, for example, is allowing the development of fast biosensing devices for point of care health and environmental applications, as can be seen in the manuscript (Wienhold et al.).
• Niwa et al. introduce the application of the TES technique for ultra-sensitive and wide-band wavelength range color imaging for biological samples with application in confocal laser scanning microscopy

The construction of novel bioluminescent fusion proteins is allowing important advances biosensing and diagnostics, for example:

• the development of intracellular calcium sensing proteins based on photoproteins with FP-tagged calcium binding domains, which allows the fast and sensitive detection of calcium flows cultured mammalian cells (Yang and Johnson) and,
• the development of a fusion protein based on a brighter firefly luciferase and ZZ-protein, which is used to detect antibodies and antigens in bioluminescent immunoassays, with sensitivity matching the commercially available chemiluminescent assays (Viviani et al.).

The development of novel brighter bioluminescent systems emitting in the FR and NIR based on combinatory chemistry and genetic engineering of luciferases, are promising for mammalian tissue bioluminescence imaging of biological and pathological processes. Among them here we report:

• A novel form of infralluciferin (Jathoul et al.) which emits in the NIR, and
• the NIR bioluminescence tracking macrophages in melanomas as shown by Zambito et al.

The engineering of luciferases is also allowing their improvement for better bioanalyitical applications such as:

• The engineering of the secreted recombinant ostracod luciferase which is allowing to better understand the structure/function relationship and to improve its expression in different heterologous systems (Mitani et al.), and
• The improvement of the thermostability of firefly luciferase which is allowing to develop more robust protein–protein interaction assays “FlimPIA” based on the functional complementation of mutant firefly luciferases (Fluc) (Ohmuro-Matsuyama et al.).

Bioluminescence is also being effectively used in biosensors for safety purposes, to detect dangerous explosives such as the optimized Escherichia coli-based bioreporter for the detection of 2,4,6-trinitrotoluene (TNT) and derivatives (DNT) (Elad et al.).

Finally, an *E.coli* bioreporter based on the randomly mutagenized ribose binding protein (RbsB) associated with the GFP-based screening was constructed to bind and detect the non-natural ligand 1,3-cyclohexanediol (13CHD) (Tavares and van der Meer).

The above papers reflect some of the continuous advances and prospects of bioengineering of bioluminescent systems and its associated detecting technologies in the fields of biomedical, environmental and safety bioanalysis.

